# First trimester secreted Frizzled-Related Protein 4 and other adipokine serum concentrations in women developing gestational diabetes mellitus

**DOI:** 10.1371/journal.pone.0242423

**Published:** 2020-11-18

**Authors:** Joost H. N. Schuitemaker, Rik H. J. Beernink, Arie Franx, Thomas I. F. H. Cremers, Maria P. H. Koster

**Affiliations:** 1 Division of Medical Biology, Department of Pathology and Medical Biology, University Medical Center Groningen, University of Groningen, Groningen, The Netherlands; 2 Research & Development, IQ Products BV, Groningen, The Netherlands; 3 Department of Analytical Biochemistry, Groningen Research Institute of Pharmacy, University of Groningen, Groningen, The Netherlands; 4 Department of Obstetrics and Gynaecology, Erasmus MC, University Medical Center Rotterdam, Rotterdam, The Netherlands; East Tennessee State University, UNITED STATES

## Abstract

**Background:**

The aim of this study was to evaluate whether soluble frizzled-related protein 4 (sFRP4) concentration in the first trimester of pregnancy is individually, or in combination with Leptin, Chemerin and/or Adiponectin, associated with the development of gestational diabetes (GDM).

**Methods:**

In a nested case-control study, 50 women with GDM who spontaneously conceived and delivered a live-born infant were matched with a total of 100 uncomplicated singleton control pregnancies based on body mass index (± 2 kg/m^2^), gestational age at sampling (exact day) and maternal age (± 2 years). In serum samples, obtained between 70–90 days gestational age, sFRP4, Chemerin, Leptin and Adiponectin concentrations were determined by ELISA. Statistical comparisons were performed using univariate and multi-variate logistic regression analysis after logarithmic transformation of the concentrations. Discrimination of the models was assessed by the area under the curve (AUC).

**Results:**

First trimester sFRP4 concentrations were significantly increased in GDM cases (2.04 vs 1.93 ng/ml; p<0.05), just as Chemerin (3.19 vs 3.15 ng/ml; p<0.05) and Leptin (1.44 vs 1.32 ng/ml; p<0.01). Adiponectin concentrations were significantly decreased (2.83 vs 2.94 ng/ml; p<0.01) in GDM cases. Further analysis only showed a weak, though significant, correlation of sFRP4 with Chemerin (R^2^ = 0.124; p<0.001) and Leptin (R^2^ = 0.145; p<0.001), and Chemerin with Leptin (R^2^ = 0.282; p<0.001) in the control group. In a multivariate logistic regression model of these four markers, only Adiponectin showed to be significantly associated with GDM (odds ratio 0.12, 95%CI 0.02–0.68). The AUC of this model was 0.699 (95%CI 0.605–0.793).

**Conclusion:**

In the first trimester of pregnancy, a multi-marker model with sFRP4, Leptin, Chemerin and Adiponectin is associated with the development of GDM. Therefore, this panel seems to be an interesting candidate to further evaluate for prediction of GDM in a prospective study.

## Introduction

Gestational diabetes mellitus (GDM) is defined as glucose intolerance that is first identified during pregnancy and, in the majority of cases, resolves after delivery. GDM is a major health problem affecting up to 10% of all pregnancies and its incidence is increasing [1]. GDM is known to be associated with a higher risk of macrosomia at birth, but also with serious long-term complications for mothers and their infants, reflecting in a significant higher risk of metabolic, including Diabetes Mellitus Type 2 (T2DM), and cardiovascular disease in later life [2–5].

However, accumulating evidence suggests that GDM is different from T2DM and that it is characterized by a mix of several pathologies with more than one potential cause, ranging from a hormonal to an autoimmune origin [6–12]. This suggests that prediction of GDM, prior to its onset, should be based on multiple markers rather than a single marker.

Screening for GDM in the first trimester of pregnancy allows prevention and intervention opportunities [13–16] and may, therefore, limit the risks of related adverse pregnancy outcome and subsequent health issues of mother and child later in life [17–22]. Currently, there are first trimester prediction models for GDM available and these are mainly based on demographic parameters [23]. However, the addition of biochemical markers might improve the performance of these prediction models [24,25]. A reason for this might be that biochemical markers are more related to the direct pathophysiology and several potential prognostic and diagnostic markers for the first and second trimester have already been described [5,26–34]. Although, none of these markers has been developed into a clinical test so far.

Adipokine markers such as Adiponectin, Chemerin and Leptin have previously been investigated in people with diabetes mellitus (DM) as well as women with GDM [35,36]. In women with GDM, Adiponectin blood concentrations are significantly decreased and this decrease is already detectable during the first trimester. For that reason, Adiponectin has been suggested as a predictive biomarker for the development of GDM [37,38]. Adiponectin is an adipocytokine abundantly present in the circulation and involved in regulating glucose concentrations as well as fatty acid breakdown [39,40]. Moreover, higher Chemerin and Leptin concentrations during early pregnancy have also been shown to be associated with the development of GDM suggesting that these two adipocytokines can also be used as predictive biomarkers of GDM [32,41]. Chemerin modulates adipogenesis and immune system functions by binding to its own receptor [42–44]. Leptin is the primary negative feedback signal from stored energy on energy intake [45]. During pregnancy elevated Leptin concentrations lead to reduced food intake [46].

Secreted Frizzled-Related Proteins modulate Wnt signalling through antagonizing the molecular pathway by a direct interaction with Wnt and they have a role in regulating cell growth, differentiation of several cell types and metabolic processes [47–49]. In mammals, this family comprises five members that are all potent inhibitors, but their other possible biological functions are less clear [50]. One of these members, Secreted Frizzled-Related Protein 4 (sFRP4), has been shown to play a role in the regulation of glucose resistance by controlling insulin secretion from islet cells [51]. With respect to this glucose resistance, sFRP4 is increased during GDM in the second trimester (i.e. ≥14 weeks) [52,53]. Interestingly, increased serum concentrations of sFRP4 were observed years *before* the diagnosis of T2DM. This strongly suggests that this protein might also be of clinical value in the first trimester for the early prediction of GDM [51,54,55]. Therefore, first trimester sFRP4 concentrations were assessed in this study to investigate the potential role of sFRP4 as an individual biomarker, as well as in combination with Chemerin, Leptin and/or Adiponectin in the development of GDM.

## Methods

### Subjects

The samples in this study were derived from a large cohort of women participating in the routine Dutch first-trimester Down syndrome screening between 2011 and 2012. As part of this screening, maternal age, gestational age (GA) at sampling, maternal weight and body mass index (BMI), method of conception, pre-existent diabetes, and smoking status were recorded by a midwife or gynaecologist. For all women, a serum sample was collected and subsequently stored at -20°C. GA was based on the fetal crown-rump length (CRL) measured during first trimester ultrasound examination using the formula of Robinson and Fleming [56]. Information about pregnancy outcomes, including pre-eclampsia and GDM, were self-reported by the participating women after delivery. All (demographic) data and anonymized samples were selected and supplied to us by the Dutch National Institute of Public Health and Environment. We did not have access to the patient records and patients actively contributed to after written informed consent. Since samples were processed anonymously without any feedback to participants, medical ethical committee approval was not necessary.

For this nested case-control study, we selected 50 serum samples drawn between 70–90 days GA from women with GDM who had no pre-existing type I or type II diabetes, who conceived spontaneously, who delivered a singleton live-born infant between 36- and 42-weeks GA, and who provided written consent for the use of spare serum for research purposes. To study crude associations and avoid potential confounding, each case was matched with two control samples from women with an uncomplicated pregnancy. Matching was based on GA (exact day), maternal age (± 2 years) and BMI (± 2 kg/m^2^).

According to standard Dutch guidelines, GDM was diagnosed based on a 75-gram oral glucose tolerance test (OGTT) when one or two of the following thresholds were exceeded: fasting glucose ≥ 7.0 mmol/L, 2 h glucose ≥ 7.8 mmol/L. An OGTT was only performed when indicated (e.g. history of GDM in an earlier pregnancy, DM in first degree relatives, development of any GDM symptoms such as excessive fetal growth on ultrasound etc.). Birth weight was adjusted for gestational age, sex and parity and converted to percentiles according to Dutch growth charts [15].

### Adipokine analysis

Adiponectin (DY1065, R&D systems, Minneapolis, MN, USA), Chemerin (DT2324, R&D systems, Minneapolis, MN, USA) and Leptin (DY398, R&D systems, Minneapolis, MN, USA) were analysed in serum using commercially available enzyme linked immunosorbent assay (ELISA) kits according to the manufacturers' instructions. Samples were diluted 1:5000 for Adiponectin, 1:500 for Chemerin and 1:250 for Leptin. When the concentration at the initial dilution was found to exceed the highest standard concentration, the sample was tested again in a tenfold higher dilution. The inter-assay and intra-assay variation were below 10% for all three assays.

The concentration of sFRP4 was also determined by ELISA (SEF878Hu, Cloud-Clone Corp., Houston, TX, USA) according to the manufacturer's instructions for use. Samples were diluted 1:50 and when the concentration at the initial dilution was found to exceed the highest standard concentration, the sample was tested again in a tenfold higher dilution. The inter-assay and intra-assay variation were 12% and 10%, respectively.

All serum samples were tested in triplicate, coded and evaluated by the investigator, who was blinded for the fact whether the sample was from an individual with a healthy pregnancy or one who developed GDM during her pregnancy.

### Statistical analysis

Baseline characteristics were expressed as median and interquartile range for continuous variables and categorical variables were expressed as numbers and percentages. Logarithmic transformation was applied for all markers to obtain normal distributions. Statistical comparisons between cases and controls were performed using Mann-Whitney U or Chi-square tests.

Univariate and multivariate logistic regression analysis was used to study the association between the marker concentrations and GDM. The discrimination of the individual marker and the multivariate model were compared by the area under the curve (AUC) of the receiver-operating curve (ROC). Additionally, correlation between the markers within the groups were assed using scatter plots and Pearson correlation coefficients. All statistical analyses were performed using SPSS (IBM version 26, Armonk, NY, USA) and p-values <0.05 were considered statistically significant.

## Results

In some cases, serum samples had to be excluded after ELISA analysis, because protein concentrations were below the lower limit of detection of the standard curve. The baseline characteristics of all cases and controls are shown in Table 1, revealing no significant differences in maternal age, maternal weight, maternal BMI, parity, and gestational age at sampling. Also, no significant differences were found in pregnancy outcomes, such as gestational age at delivery and birth weight.

**Table 1:** Baseline characteristics of pregnancies affected by gestational diabetes (GDM) and uncomplicated pregnancies (controls).

Variable	GDM	Controls	p-value
	(n = 50)	(n = 100)	
Maternal age (years)	35.0	34.8	0.678^#^
	(31.8–37.3)	(30.8–37.6)	
Maternal weight (kg)	75	75	0.625^#^
	(67–88)	(64–85)	
Maternal BMI (kg/m^2^)	26.6	26.2	0.802^#^
	(22.6–30.1)	(22.3–29.6)	
Smoking	0	0	1.000^##^
	(0%)	(0%)	
Parity	1	1	0.083^#^
	(1–2)	(0–2)	
Gestational age at sample collection (days)	83	83	1.000^#^
	(75–86)	(75–86)	
Gestational age at delivery (days)	281	281	0.417^#^
	(273–284)	(275–286)	
Fetal gender (male)	27	55	0.908^##^
	(54%)	(55%)	
Birth weight (g)	3718	3530	0.230^#^
	(3310–3980)	(3283–3875)	
Birth weight percentile	65	49	0.180^#^
	(37–79)	(31–74)	

Maternal age, weight, BMI and smoking were assessed at sample collection (first trimester of pregnancy). Data are presented as median and interquartile range for continuous and N (%) for categorical variables.

^#^ analysis by Mann-Witney U test and

^##^ analysis by Chi-square test.

**Fig 1:**
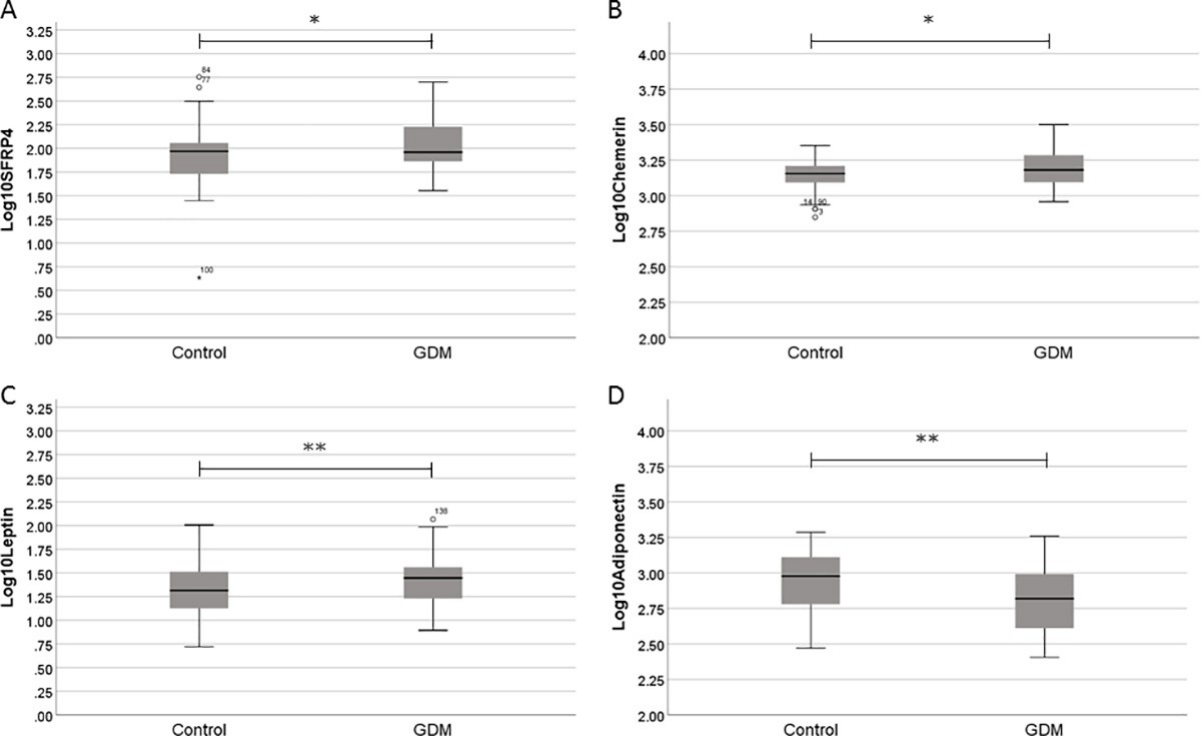
Boxplots of uncomplicated (control) pregnancies or pregnancies that will develop gestational diabetes (GDM). Median, 25%-75% percentiles (box) and 5%-95% percentiles (whiskers) of sFRP4 (A), Chemerin (B), Leptin, (C) and Adiponectin (D) log10 transformed concentrations. * = p<0.05; ** = p<0.01.

Logarithmic transformed sFRP4 serum concentrations in GDM pregnancies were 2.04 ± 0.25 ng/ml and this was significantly higher than the control pregnancies (1.93 ± 0.28 ng/ml (p<0.05), Fig 1A). Additionally, Chemerin and Leptin were significantly increased in GDM with 3.19 ± 0.13 ng/ml vs 3.15 ± 0.09 ng/ml for Chemerin (p<0.05, Fig 1B) and 1.44 ± 0.28 ng/ml vs 1.32 ± 0.24 ng/ml for Leptin (p<0.01, Fig 1C). On the contrary, Adiponectin concentrations were significantly decreased in GDM with 2.83 ± 0.23 ng/ml vs 2.94 ± 0.20 ng/ml (p<0.01, Fig 1D).

Univariate logistic regression analysis of the logarithmic transformed data showed statistically significant odds ratios (OR) for all markers: 5.24 (95%CI 1.29–21.3) for sFRP4, 40.1 (95%CI 1.53–1050) for Chemerin, 6.51 (95%CI 1.61–26.4) for Leptin and 0.09 (95%CI 0.02–0.49) for Adiponectin (Table 2A). Therefore, all markers were subsequently included in a multivariate logistic regression model (Table 2B). In this combined model, only Adiponectin showed a statistically significant OR (0.12 (95%CI 0.02–0.68)).

**Table 2:** Logarithmic transformed mean concentrations and standard deviation (SD) and logistic regression analysis of sFRP4, Chemerin, Leptin and Adiponectin concentrations in gestational diabetes (GDM) and uncomplicated (control) pregnancies.

*A*. *Univariate logistic regression analysis*				
**Markers**	**Mean concentration ±SD**		**OR (95%CI)**		**p-value**
	**(ng/ml)**				
	Control	GDM			
	(n = 100)	(n = 50)			
sFRP4	1.93 ± 0.28	2.04 ± 0.25	5.24 (1.29–21.27)	0.020
Chemerin	3.15 ± 0.09	3.19 ± 0.13	40.10 (1.53–1049.6)	0.027
Leptin	1.32 ± 0.24	1.44 ± 0.28	6.51 (1.61–26.44)	0.009
Adiponectin	2.94 ± 0.20	2.83 ± 0.23	0.09 (0.02–0.49)	0.005
*B*. *Multivariate logistic regression analysis*				
**Markers**			**OR (95%CI)**	**p-value**
sFRP4			2.15 (0.43–10.68)	0.350
Chemerin			12.83 (0.34–491.54)	0.170
Leptin			2.90 (0.59–14.14)	0.189
Adiponectin			0.12 (0.02–0.68)	0.017

**Fig 2:**
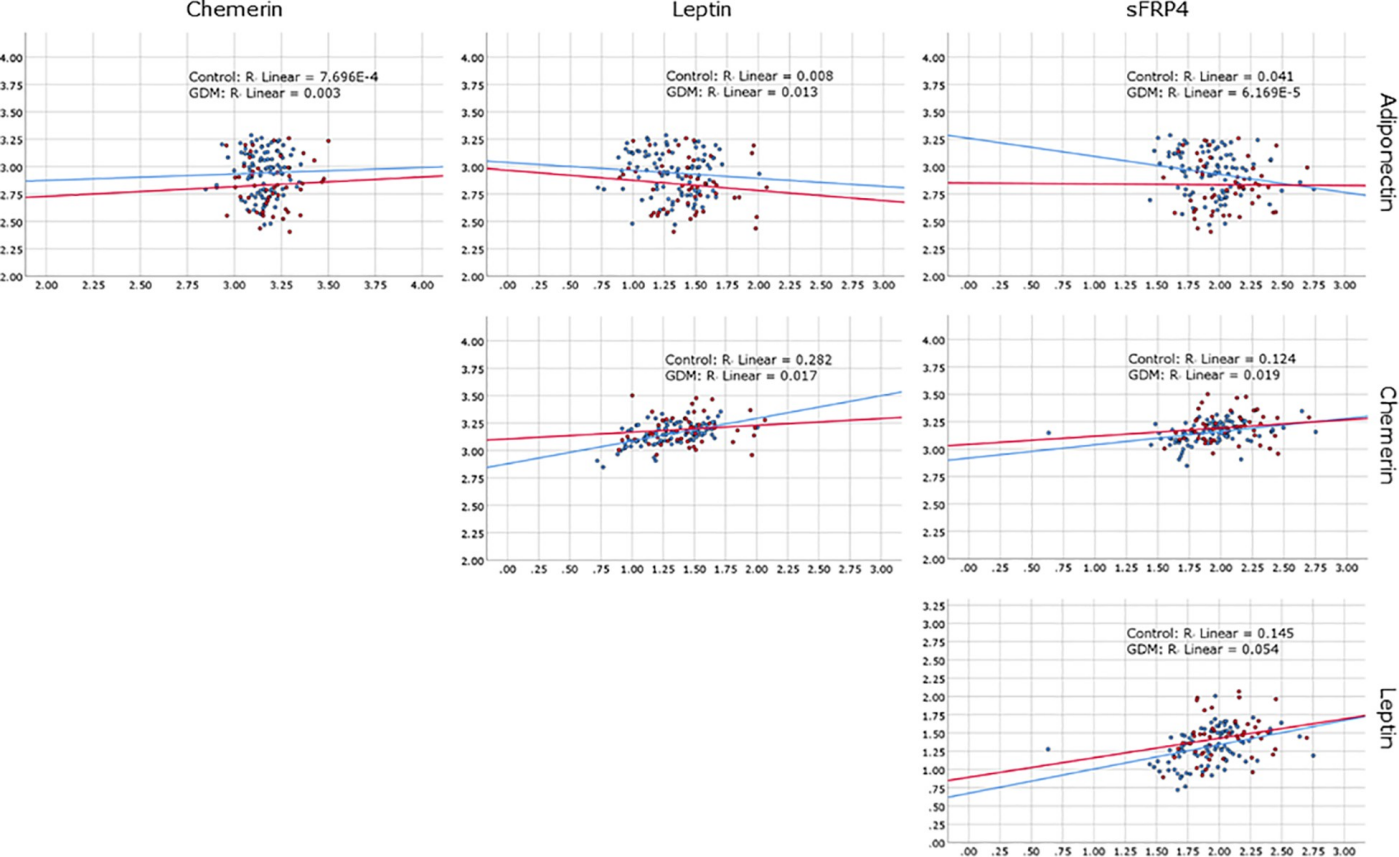
Correlation plots of Adiponectin, Chemerin, Leptin and sFRP4 log10 transformed concentrations in GDM and control pregnancies.

Correlation between the markers within the control group were weak, but statistically significant for sFRP4 with Chemerin (R^2^ = 0.124, p<0.001), sFRP4 with Leptin (R^2^ = 0.145, p<0.001) and Chemerin with Leptin (R^2^ = 0.282, p<0.001). There was *no* statistically significant correlation between markers within the GDM group (Fig 2 and Table 1A and 1B in S1 Table).

**Fig 3:**
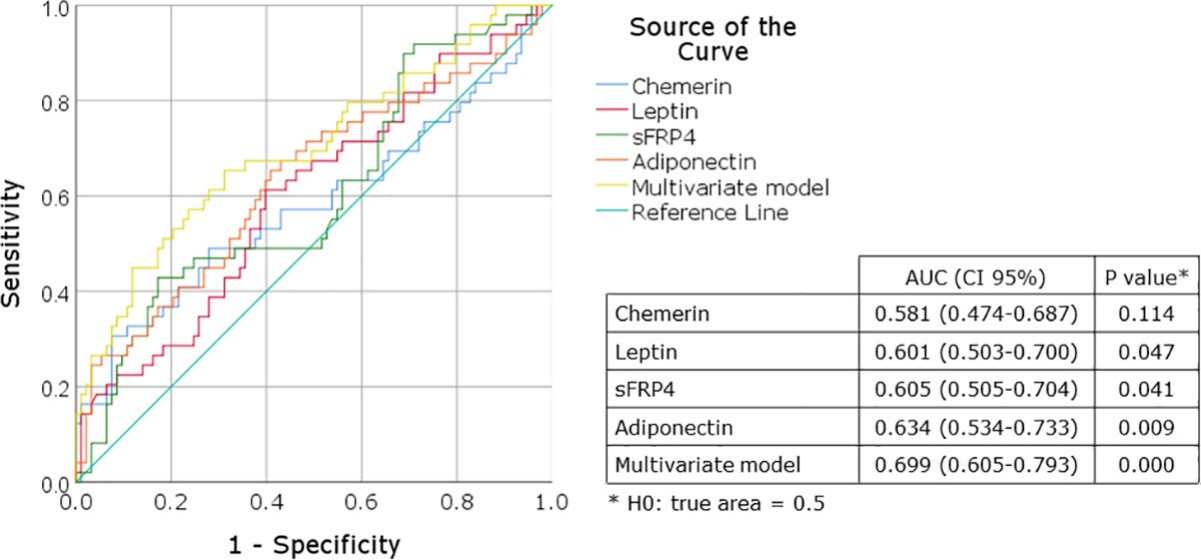
Receiver-operating curve and logistic regression data for sFRP4, Chemerin, Leptin, Adiponectin and multivariate model. AUC, area under the receiver-operating curve.

The discriminative performance of the univariate models, expressed as AUC, were 0.581 (95%CI 0.474 to 0.687) for Chemerin, 0.601 (95%CI 0.503 to 0.700) for Leptin, 0.605 (95%CI 0.505 to 0.704) for sFRP4 and 0.634 (95%CI 0.534 to 0.733) for Adiponectin. Discriminative performance increased when all biomarkers were combined in a multivariate model (AUC 0.699 (95%CI 0.605 to 0.793, Fig 3).

## Discussion

To the best of our knowledge, this is the first study reporting the association of increased sFRP4 concentrations with the development of GDM in the first trimester. Additionally, we show that combining sFRP4, Leptin, Chemerin and Adiponectin in a multivariate model gives a good discrimination for GDM in the first trimester.

Increased concentrations of sFRP4 in first trimester samples of women who later developed GDM could be explained by the fact that the sFRP4 gene was identified in a systems genetic study as one of the genes associated with pancreatic islet dysfunction [57]. Furthermore, it was reported that sFRP4 is overexpressed in pancreatic islet cells of patients with T2DM, while its increased circulatory concentrations impair insulin secretion by β-cells and thereby reduce glucose tolerance [51]. These findings point towards a direct role of sFRP4 in controlling the insulin secretion from β-cells. In addition to its effect on glucose metabolism, elevated concentrations of sFRP4 also impair triglyceride metabolism in patients with T2DM [54]. Despite the clear link between sFRP4 and T2DM, an association between sFRP4 and GDM has only been reported in the second trimester or later, and prior to this study not yet in the first trimester. The phenotypic onset of GDM happens in the second trimester or later and, therefore, measurement of markers in the second and third trimester shows more predictive power. However, sooner diagnosis is better, since a timely therapeutic intervention is essential to prevent the adverse pregnancy outcome. Described higher concentrations of sFRP4 during GDM in the second trimester support our suggested association in the first trimester. The biological origin of a higher chance to develop GDM, when sFRP4 serum concentrations in the first trimester are increased, is as yet unclear and remains to be investigated further. However, another member of the Wnt antagonists, sFRP5, has also been associated with GDM and T2DM [58–60]. This link between GDM and two members of the sFRP protein family implicates a role of the Wnt signaling pathway in impaired glucose tolerance. Furthermore, our results are in concordance with previous studies that showed decreased Adiponectin concentrations [37,38,61] and increased Chemerin and Leptin concentrations [32,36,41,62] in the first trimester of pregnancy in women who later developed GDM.

In contrast to T2DM, it is proposed that GDM has a hormonal and autoimmunological background as underlying pathological mechanism [6–12]. This crosstalk scenario, fuelled by adipokines, plays an important role in metabolic homeostasis under healthy conditions, whereas a release of proinflammatory adipocytokines is important in the pathogenesis of many metabolic disorders. Our panel of sFRP4, Adiponectin, Leptin and Chemerin confirms this crosstalk scenario of GDM. This study was limited to four biomarkers and it is therefore conceivable that parallel evaluation of more hormonal and inflammatory GDM-associated markers, like sex hormone-binding globulin, C-reactive protein and/or sFRP5 [30,58,63,64], would strengthen our initial observation and thereby validate the use of a panel, including sFRP4, to predict the risk on GDM.

We have been able to select pregnancies with comparable and normal gestational age at delivery, birth weight and limited adverse outcome. Although one might expect shorter pregnancy durations and higher birth weights in GDM pregnancies, due to accurate screening and monitoring of GDM pregnancies in the Netherlands, the number of adverse outcomes in these pregnancies are relatively low [65,66]. Nevertheless, women who went through a GDM pregnancy are still at considerably higher risk to develop T2DM later in life [67]. Since the cases and controls were matched on potentially predictive variables such as BMI, it was not possible to evaluate the added value of sFRP4 concentrations on top of these demographic baseline predictors.

The association of increased sFRP4 concentrations with the development of GDM was observed in a cohort with a relatively low BMI (26.4 (± 5.2) kg/m^2^). The relatively low BMI might indicate that our conclusions are drawn on pregnant women who are, based on current prediction models, considered as low-risk GDM cases, since BMI is one of the major demographic baseline predictors [23]. Moreover, one of the major outcomes of GDM is an increased fetal birth weight, but this was absent in our cohort [2,3]. Unfortunately, it is not possible to say anything about the severity of the GDM cases or to conclude that only mild GDM cases were included in this study, since the database did not have any information on (therapeutic) interventions which might have influenced the fetal birth weight. Additionally, the absence of more data of the individual women made it impossible to speculate what the actual cause of GDM might have been. More significant observations might be obtained in an obese cohort (BMI of ≥30 kg/m^2^), or GDM cases with an increased fetal birth weight/percentile.

This study also has some limitations. Firstly, the data were obtained from a relatively small number of samples and it would be desirable to extend the observations of this study by using a larger number of samples from an unselected and mixed population and preferably as a prospective study. This will give the opportunity to critically validate the data of this discovery study. Secondly, information on pregnancy outcome, including GDM, was collected through self-reporting by the participating women. This may have led to classification bias, however, previous studies based on this specific cohort have shown that self-reporting of pregnancy outcomes shortly after pregnancy is a reliable method [68,69]. Thirdly, within the Dutch obstetric care system it is standard policy to perform an OGTT only when indicated (e.g. based on risk factors for GDM or excessive fetal growth observed on ultrasound). This might also have led to classification bias of the diagnosis and thus a possible underestimation of GDM in the control group.

Nevertheless, we have shown in this study that an increased concentration of serum sFRP4 in the first trimester of pregnancy is associated with a higher risk to develop GDM later in pregnancy and that including sFRP4 in a multi-marker model with Leptin, Chemerin and Adiponectin gives a fair discrimination for GDM. Current prediction models in the first trimester of pregnancy are mainly based on demographic parameters and their predictive power can be improved when a biochemical marker is included. Validating sFRP4 in combination with demographic parameters and the markers Adiponectin, Leptin and Chemerin in a prospective cohort might increase the early prediction of GDM in the first trimester.

## Supporting information

S1 Table Pearson’s correlations for uncomplicated (control) pregnancies (S1A Table) and gestational diabetes (GDM) pregnancies (S1B Table). (DOCX)

S2 Table Dataset with cohort characteristics and biomarker data. (XLSX)
