# Supplementary material for: First trimester secreted Frizzled-Related Protein 4 and other adipokine serum concentrations in women developing gestational diabetes mellitus
Source: PLoS One. 2020 Nov 18;15(11):e0242423. doi: 10.1371/journal.pone.0242423 (PMC7673552; doi:10.1371/journal.pone.0242423)
Supplement: S1 Table — (DOCX) [file pone.0242423.s001.docx]

**S1 Table. Pearson’s correlations for uncomplicated (control) pregnancies (Table 1A) and gestational diabetes (GDM) pregnancies (Table 1B).**

*A. Pearson’s correlations (2-tailed) between log10 transformed sFRP4, Chemerin, Adiponectin, and Leptin concentrations for all uncomplicated (controls) pregnancies*

|  |  | **sFRP4** | **Chemerin** | **Adiponectin** | **Leptin** |
| --- | --- | --- | --- | --- | --- |
| **sFRP4** | Pearson Correlation | 1 | 0.353^**^ | -0.202 | 0.381^**^ |
|  | p-value |  | 0.000 | 0.052 | 0.000 |
|  | N | 96 | 96 | 93 | 96 |
| **Chemerin** | Pearson Correlation | 0.353^**^ | 1 | 0.028 | 0.531^**^ |
|  | p-value | 0.000 |  | 0.790 | 0.000 |
|  | N | 96 | 98 | 95 | 98 |
| **Adiponectin** | Pearson Correlation | -0.202 | 0.028 | 1 | -0.088 |
|  | p-value | 0.052 | 0.790 |  | 0.398 |
|  | N | 93 | 95 | 95 | 95 |
| **Leptin** | Pearson Correlation | 0.381^**^ | 0.531^**^ | -0.088 | 1 |
|  | p-value | 0.000 | 0.000 | 0.398 |  |
|  | N | 96 | 98 | 95 | 98 |
| *p-value <0.05 (2-tailed) | | | | | |
| **p-value <0.01 (2-tailed) | | | | | |

*B. Pearson’s correlations (2-tailed) between log10 transformed sFRP4, Chemerin, Adiponectin, and Leptin concentrations for all gestational diabetes (GDM) pregnancies*

|  |  | **sFRP4** | **Chemerin** | **Adiponectin** | **Leptin** |
| --- | --- | --- | --- | --- | --- |
| **sFRP4** | Pearson Correlation | 1 | 0.138 | -0.008 | 0.232 |
|  | p-value |  | 0.343 | 0.957 | 0.109 |
|  | N | 49 | 49 | 49 | 49 |
| **Chemerin** | Pearson Correlation | 0.138 | 1 | 0.051 | 0.131 |
|  | p-value | 0.343 |  | 0.726 | 0.363 |
|  | N | 49 | 50 | 50 | 50 |
| **Adiponectin** | Pearson Correlation | -0.008 | 0.051 | 1 | -0.112 |
|  | p-value | 0.957 | 0.726 |  | 0.437 |
|  | N | 49 | 50 | 50 | 50 |
| **Leptin** | Pearson Correlation | 0.232 | 0.131 | -0.112 | 1 |
|  | p-value | 0.109 | 0.363 | 0.437 |  |
|  | N | 49 | 50 | 50 | 50 |
| *p-value <0.05 (2-tailed) | | | | | |
| **p-value <0.01 (2-tailed) | | | | | |
